# Loneliness and social isolation causal association with health-related lifestyle risk in older adults: a systematic review and meta-analysis protocol

**DOI:** 10.1186/s13643-019-0968-x

**Published:** 2019-02-07

**Authors:** Martin Malcolm, Helen Frost, Julie Cowie

**Affiliations:** 1 School of Health Sciences, University of Stirling, FK9 4LA Scotland, UK; 2 School of Health and Social Care, Edinburgh Napier University, Room 3.B.45, 9 Sighthill Court, Edinburgh, EH11 4BN UK; 3 NMAHP Research Unit, Glasgow Caledonian University, 70 Cowcaddens Road, Glasgow, UK

**Keywords:** Loneliness, Social isolation, Health behaviours, Alcohol, Smoking, Obesity, Physical activity, Systematic review, Meta-analysis, Older adults

## Abstract

**Background:**

The health impacts of loneliness and social isolation among older adults are widely acknowledged. Despite this, there is no consensus on the possible causal nature of this relationship, which could undermine effectiveness of interventions. One body of thought is that loneliness and social isolation affect health-related behaviours to indirectly damage health. However, there has not been any systematic assessment of the association between loneliness and social isolation and health-related behaviours which considers the possible impact from confounding factors and the causal direction of this association.

**Methods/design:**

The research will comprise a systematic review and meta-analysis to address the evidence gap. EMBASE, MEDLINE, PSYCINFO, CINAHL, SocIndex, Scopus and Web of Science will be systematically searched for quantitative observational studies considering an association between loneliness/social isolation and key health-related behaviours in older adults. Two reviewers will independently check the study titles and abstracts for eligibility. Included studies will be critically appraised using Newcastle-Ottawa Scale by the lead author and checked by the second reviewer. Discrepancies in eligibility or quality assessment will be resolved via discussion or referral to a third reviewer. Results will be synthesised and reported in accordance with the Centre for Reviews and Dissemination (CRD) guidelines. This will be in the form of a descriptive summary, risk of bias assessment together with a meta-analysis and sub-group analyses (for covariate adjusted results) where sufficient heterogeneity of results is established. Finally, any associations identified will be analysed using the Bradford-Hill criteria to explore causal relationships which, if they exist, will be reported by means of a computed causations score.

**Discussion:**

This review aims to assess the extent and causal nature of associations between loneliness/social isolation and health-related behaviours among older adults. This data will provide a comprehensive overview of the quality of the evidence base to inform stakeholders in tackling the growing public health challenges arising from loneliness/social isolation in ageing populations.

**Systematic review registration:**

PROSPERO CRD42017020845

**Electronic supplementary material:**

The online version of this article (10.1186/s13643-019-0968-x) contains supplementary material, which is available to authorized users.

## Background

### Loneliness/social isolation and ill-health

The health impacts of loneliness and social isolation are widely recognised. Evidence of their adverse impacts on mental health is particularly strong, including outcomes such as depression [1–7], anxiety [8], schizophrenia [9, 10], suicide [11–14] and dementia and Alzheimer’s disease [15–17]. The link with physical disease has been made between elevated risk among lonely or isolated people and coronary heart disease and cardiovascular conditions [18–21]. In addition, there is evidence of a significant association between loneliness and cancer [22] as well as greater susceptibility to infectious diseases [23, 24].

Although distinct concepts, loneliness and social isolation are often used interchangeably and therefore both need to be considered together in examining their health impacts. Loneliness is a perceived deficit between actual and desired quality or quantity of relationships [25]. Social isolation is the objectively quantified shortfall in an individual’s social relationships often measured in terms of social network size, diversity or frequency of contacts [26]. People can be socially isolated without feeling lonely, or feel lonely despite having an adequate quantity of social relationships.

Despite their acknowledged health impacts, both loneliness and social isolation prevalence rates have persisted over several decades [27]. With levels greatest among older people linked to loss of contemporaries, cognitive decline, disability and the loss of social roles [28–30], Consequently, much of the research to date on the adverse health impact from loneliness/social isolation has focused on older adults particularly for age-related health conditions [31, 32]. In addition, a number of longitudinal studies [1, 3–5, 15, 33] have shown the cumulative effects of loneliness/social isolation on ill-health over time thereby manifesting greatest among older adults, particularly the oldest old who receive greatest cumulative exposure [29, 34, 35]. Exacerbating these health impacts on older adults are several socio-demographic trends in recent decades. The first has been the dramatic rise in chronic long-term conditions to become the main source of morbidity and mortality among older adults. Typically, long-term conditions are associated with loneliness [1–11, 15–22]. Secondly, the ageing population in many countries means that there are increasing numbers of older adults living longer and at greater risk of exposure to loneliness/social isolation and their associated ill-health effects [36, 37]. Finally, increasing proportions of older adults living in single person households means that greater proportions are likely to experience loneliness and isolation which are strongly correlated with living alone [28].

The combination of socio-demographic trends and the health impacts of social isolation/loneliness has made this a major public health concern [38]. There is strong evidence of social isolation/loneliness having greater or equivalent risk to mortality than smoking or obesity respectively [39–43]. In response, governments in the UK are formulating national strategies [44–46] to tackle the effects while health and social care services have new policy commitments [47–49] in recognition of growing service costs from loneliness/social isolation [50]. With such governmental focus on loneliness/social isolation and preventing associated ill-health outcomes, there is an urgent need to improve our understanding of how the relationship between loneliness/social isolation and health operates to effectively intervene for those most likely to be affected.

### Evidence on loneliness/social isolation and health in older adults and current gaps

Despite demographic and social trends and associated health impacts, there remain significant gaps in our understanding of the link between loneliness/social isolation and ill-health. Few studies investigate the indirect ill-health pathways via health-related behaviours and instead focus on direct biological or physiological pathways [23, 51–58]. Of those studies which do consider the association with health-related behaviours some suggest loneliness/social isolation is associated with lower physical activity [59], alcohol misuse [60] and smoking [53, 61] while others found no evidence of variation in lifestyle behaviours between lonely and non-lonely older people [23, 52, 54]. This led some to dispute the claim that health behaviours are associated with mortality and morbidity among lonely persons [62]. Added to this gap in evidence on health-related behaviours, causal link with loneliness/social isolation among older people is the role of potential confounding or effect modifying covariates, e.g. poverty and low educational attainment [63–66]. This may have important consequences in shaping effective public health interventions.

### Review literature

Previous reviews on social relationships causal associations with ill-health have not employed clear definitions or included objective and broader measures of functional social support, social integration or social capital [28, 39, 40, 42, 43] or other related measures, e.g. living alone [67–69] or marital status [70–72]. Many reviews on social isolation or loneliness do not address the evidence for a causal relationship with ill-health [11, 33, 43, 50–52, 73–75]. Of the few reviews which have investigated the causal pathways, most have used a discursive theoretical approach rather than a systematic synthesis of empirical data or given limited examination of behavioural pathways [51, 52, 58]. Two reviews have specifically considered the potential indirect health risk due to lifestyle factors but these are either out of date, not systematic, not focused on older people or consider only a single health behaviour [60, 76]. In addition, only a few reviews have focused on older adults and ill-health despite prevalence of loneliness/social isolation being greatest in this group [33, 75, 77].

### Aims and objectives

The aim of this study will be to synthesise and assess the evidence of the extent and nature of any association between loneliness and/or social isolation and health-related lifestyle behaviours risk including the extent due to other related factors. This will lead to a better understanding for the development of targeted and effective interventions. The key objectives are:

1. To establish the extent of association between loneliness or social isolation and key health-related behaviour risks, specifically alcohol or drugs misuse, smoking, physical activity and obesity.
2. To assess the evidence for any causal relationship in any associations found.
3. To report whether any association found between loneliness and/or social isolation and health-related behaviours is independent of known covariates.

## Methods and design

The design of this research will be a systematic review. This systematic review will be carried out in accordance with the reporting guidelines and checklist of criteria set in Preferred Reporting Items for Systematic Review (PRISMA) [78] and if appropriate the Meta-analysis of Observational Studies in Epidemiology (MOOSE) guidance for systematic review reporting.

### Inclusion criteria

#### Study types

The review will include any interventional or observational study that quantitatively assesses the associations of loneliness/social isolation and selected health-related behaviours and the evidence of causation. Cross-sectional studies will be included for assessing associations but not causality. No date restrictions will be placed upon search. For mixed methods studies, only the quantitative element will be extracted and reviewed where it meets the inclusion criteria.

#### Population type

Studies involving older adult aged 50 and older. There will be no restrictions on any other participant characteristics.

### Exposure measures

Studies reporting clearly defined loneliness and/or social isolation exposure measures. For the purposes of this review loneliness will be defined as a perceived deficit between the actual and desired quantity or quality of an individual’s social relationships while social isolation is an objectively measured shortfall in the level of an individual’s social contacts [25, 26]. A number of such measures have been utilised in the literature from single item to multi-dimensional scales [79–82]. Some studies interchange the terms loneliness and social isolation and an assessment will be made to allocate the exposure to the correct type.

### Outcome measures

Studies including at least one key health-related risk behaviour as the outcome of interest, e.g. tobacco use, alcohol misuse, physical activity or obesity, since these are acknowledged as the primary lifestyle risk factors with a causal association upon many chronic health conditions associated with loneliness/social isolation [83, 84].

### Covariates

Studies including socio-demographic and ill-health covariates on identified associations. Studies that do not contain estimates adjusted for these covariates will be included but with potentially confounded results noted.

### Exclusion criteria

Studies that do not test for empirical associations between loneliness or social isolation and the specified health-related lifestyle behaviours will be excluded.

Studies utilising proxy measures of loneliness or social isolation such as ‘living alone’ or ‘marital status’ or wider measures such as social relationships or social support will be excluded since these do not represent the specific exposures of interest.

Non-English language studies will be excluded though no geographical restrictions will be applied.

### Identification of eligible studies and data extraction

#### Search strategy

The following databases and electronic collections will be searched for relevant publications: OVID MEDLINE, OVID Nursing, EMBASE, CINAHL, PsychINFO, SocINDEX (Gerontology, Psychology, Social Sciences and Sociology categories), Social Work Online, Scopus and Web of Science. Manual hand searching of reference lists from identified studies will be undertaken for any overlooked articles or dissertations of relevance. In addition, the search will include the grey literature available from the above specified databases with no restriction made on publication type. Further grey literature will be identified via the searching of targeted websites of health organisations, older persons agencies and relevant campaigning organisations (e.g. Campaign for Loneliness). Key content experts will be contacted directly to identify any further documents (e.g. via the Campaign for Loneliness Research Hub membership). The full description of the search terms and search strategy devised for MEDLINE database is provided (see Additional file 1), which will be adapted as required for use in searching other databases.

The search strategy will use both Medical Subject Heading (MesH) terms (MEDLINE and CINAHL) and Major Subject Headings (PsychINFO) as well as keyword searching on exposure and outcomes of interest (all databases). The sensitivity and specificity of search will be maximised by use of explosion and truncating of subject terms and Boolean searching on synonyms of loneliness and the key health-related behaviours. The strategy will be reviewed by an experienced librarian to assess its quality based on Peer Review of Electronic Search Strategy (PRESS) Tier 1 checklist elements [85].

#### Selection of studies

The selection of studies will follow the checklist contained in the PRISMA guidelines [86]. The lead reviewer (MM) will independently screen all retrieved study titles and abstracts to assess eligibility against the inclusion/exclusion criteria and import into RefWorks before removing any duplicates. A second reviewer will independently assess the accuracy of all screened study titles. Any discrepancies between reviewer selections will be resolved by discussion between reviewers or on reference to a third reviewer if consensus is not achieved. Full texts of potentially eligible studies will be obtained and reviewed by the lead reviewer where required to support screening of studies for final inclusion. A second reviewer will assess all potentially eligible full texts for confirming final included studies. For any papers where there is uncertainty regarding inclusion the paper will be reviewed by a third reviewer. Study authors will be contacted should clarification be required. The rationale for exclusion will be recorded as part of the screening process and reported in accordance with the stated eligibility criteria. The PRISMA search flow chart will be used to record the number of studies included and excluded at each step in the process.

#### Data extraction

Data extraction will be performed by the lead reviewer and checked by two secondary reviewers using a pre-defined data extraction form created in Excel and based upon Centre for Reviews and Dissemination (CRD) guidelines [87] and the Joanna Briggs Institute Data Extraction Form for Prevalence and Incidence Studies [88]. Variables will be extracted for the following key groupings: general study information, study characteristics, participant characteristics, exposure measures, outcome data and analysis/results (see Additional file 2 .

#### Study quality and critical appraisal

The quality of eligible studies will be appraised using the Newcastle-Ottawa Scale (NOS) [89], a tool developed specifically for assessing quality of observational epidemiological studies. Versions of the NOS tool for both case control and cohort studies will be used together with an adapted version for cross-sectional studies. The NOS assesses study bias through star rating according to selection of study groups, comparability of the groups and ascertainment of either exposure being tested for case control studies or outcome of interest for cohort studies. This assessment of quality will be undertaken by the first author and checked independently for completeness and accuracy by a second author. Any differences in the assessment of quality will be resolved via discussion between these authors and if remains unresolved upon referral to a third author.

#### Synthesis and analysis

The data will be categorised into each health-related behaviour outcome and loneliness or social isolation exposure type. Data from eligible studies will first be synthesised as per CRD ‘Systematic Reviews: CRD Guidance for undertaking reviews in healthcare, 2009’ guidance by means of a descriptive summary in tabular format concerning study type, exposure, participant characteristics and outcome measures. Secondly, a summary table on the quality of studies will be provided according to three potential categories of risk bias identified using the NOS tool, specifically selection, comparability and exposure/outcome.

Meta-analysis will be considered depending on the availability of data of sufficient quality and similarity for each of the exposures and health-related behaviours. As indicated by the Cochrane Handbook for Systematic Reviews [90] recommendation, the extent of heterogeneity will be assessed using the *I*^2^ statistic. Meta-analysis of studies will be carried out on pooled results where *I*^2^ < 60% using a fixed or random effects model as appropriate [90].

Individual study results (odds ratios, risk ratios, prevalent ratios) will be reported along with 95% confidence intervals (CIs). If sufficient homogeneity is identified, then results will be converted to odds ratios (ORs) where required and reported as combined ORs. Pooled ORs will be reported for the effect of loneliness/social isolation upon each category of health behaviour. Sub-group analysis will be performed on studies providing adjusted results where there are a sufficient number of studies to permit pooling of results at this level. This will be performed on groups of either partially adjusted results comprising up to two covariates (normally age and gender) or fully adjusted results for greater than two covariates. To assess for potential publication bias, funnel plots of effect estimates against sample sizes will be produced and examined for their symmetry.

A narrative synthesis will be completed if there is insufficient number, quality or similarity of data to permit a formal meta-analysis or sub-group analysis. This will follow best practice guidance on conducting narrative synthesis [91] to summarise the current state of knowledge and describe the study designs, the findings and the robustness of the evidence including the strengths and limitations of studies and the conclusions drawn from the results. This will include assessment for publication bias via a funnel plot of sample size v. effect size should there be a high proportion of studies with significant findings. The overall strength of the synthesised evidence will be assessed using GRADE (Grades of Recommendation, Assessment, Development, and Evaluation) criteria [92].

#### Assessment for causality

Each outcome-exposure category will be analysed according to applicable Bradford Hill criteria of causation [93] to assess whether any association between loneliness or social isolation and each health-related behaviour identified in the synthesis and analysis has a potential causal relationship. The assessed Bradford Hill criteria will include:

1. Temporality
2. Association (strength and significance of association)
3. Dose-response relationship (or biological gradient)
4. Consistency

Other Bradford Hill criteria for causality will not be applied: ‘experiment’, since there is an absence of empirical/RCT research in this area; ‘specificity’, since loneliness/social isolation have several possible outcomes; ‘coherence’, given this is usually tested by conformity of surrogate outcomes to the pathology of the ill-health outcome observed. In this review, the key lifestyle behaviours are risk factors of ill-health and so surrogates by definition; ‘plausibility’, since associations observed would be expected to have probable mechanisms posited by researchers as explanation; ‘analogy’ since alternative similar associations (e.g. from social support deficits or depression) that may be applied as analogous arguments to support causation do not assist in the key aim of this review, which is to specifically isolate the particular psycho-social construct of loneliness and objective social isolation and their causal association with the outcomes of interest.

The results from each study will be summarised to determine the overall levels of evidence for each criterion of causality for each loneliness and health behaviour category. This will be carried out by means of a causation score for each health-related behaviour and loneliness/social isolation category. The score is calculated according to the unweighted sum of the number of criteria met where a score of 4 is adjudged to be indicative of strong evidence of cause and effect relationship, a score of three to show moderate level of evidence and two or lower to indicate weak evidence [94, 95].

## Discussion

This will be the first systematic review specifically focused on associations and potential causal relationships between loneliness/social isolation with health-related behaviours among older adults. In addition, it will seek to assess the role of a range of socio-demographic factors on this association. The strengths of the systematic review include clear definitions and inclusion criteria, transparent systematic approach to searching, screening, assessing and extracting which utilises standardised forms and independent review wherever possible. The systematic review will be strengthened by the appropriate use of standard reporting instruments such as PRISMA, NOS, and GRADE. Additionally, reporting will be structured and comprehensive, including critical appraisal, narrative synthesis and, if appropriate, meta-analysis.

It is timely for such a review given the growing prominence of the issue in the media [96–98] and health policy [45–49]. Such growing media and government attention recognises the socio-demographic trends fuelling loneliness and social isolation among older adults to the extent it has become an acknowledged public health challenge. Therefore, understanding the association and causal pathways between loneliness/social isolation and ill-health will be important for developing interventions and strategies to combat loneliness and social isolation.

## Additional files

Additional file 1: Medline Search Strategy. File contains the Medline search strategy syntax used in the review including keywords and Mesh subject headings which will be adapted for other databases included within the systematic review. (DOCX 29 kb)

Additional file 2: Date Extraction Form Variables. File contains the details on the variables and their groupings to be extracted and recorded on the study data extraction form for the systematic review (DOCX 13 kb)

## Abbreviations

CIs: Confidence intervals
CRD: Centre for Reviews and Dissemination
GRADE: Grades of Recommendation, Assessment, Development and Evaluation
NOS: Newcastle-Ottawa Scale
ORs: Odds ratios
PRESS: Peer Review of Electronic Search Strategy
PRISMA: Preferred Reporting Items for Systematic Review
