# Supplementary material for: Loneliness and social isolation causal association with health-related lifestyle risk in older adults: a systematic review and meta-analysis protocol
Source: Syst Rev. 2019 Feb 7;8:48. doi: 10.1186/s13643-019-0968-x (PMC6366024; doi:10.1186/s13643-019-0968-x)
Supplement: Supplementary file 1 — Medline Search Strategy. File contains the Medline search strategy syntax used in the review including keywords and Mesh subject headings which will be adapted for other databases included within the systematic review. (DOCX 29 kb) [file 13643_2019_968_MOESM1_ESM.docx]

Medline Search strategy

| 1. | exp Alcoholism/ |  |
| --- | --- | --- |
| 2. | exp Alcoholic Intoxication/ |  |
| 3. | exp Alcohol Drinking/ |  |
| 4. | exp Substance-Related Disorders/ |  |
| 5. | exp Alcohol-Related Disorders/ |  |
| 6. | (alcohol* or binge drink* or (drug adj2 abus*) or (substance adj2 abus*) or (drug adj2 misus*) or (drugs adj2 misus*)).mp. [mp=title, abstract, original title, name of substance word, subject heading word, keyword heading word, protocol supplementary concept word, rare disease supplementary concept word, unique identifier] |  |
| 7. | 1 or 2 or 3 or 4 or 5 or 6 |  |
| 8. | exp "Tobacco Use"/ or exp "Tobacco Use Disorder"/ or exp Tobacco/ or exp Tobacco Products/ |  |
| 9. | exp Smoking/ |  |
| 10. | (smoking or smokers or tobacco or nicotine).mp. [mp=title, abstract, original title, name of substance word, subject heading word, keyword heading word, protocol supplementary concept word, rare disease supplementary concept word, unique identifier] |  |
| 11. | 8 or 9 or 10 |  |
| 12. | exp Exercise Tolerance/ or exp Exercise/ or exp Exercise Therapy/ |  |
| 13. | exp Motor Activity/ |  |
| 14. | exp Physical Exertion/ |  |
| 15. | (physic* activ* or physic* fit* or exercis*).mp. [mp=title, abstract, original title, name of substance word, subject heading word, keyword heading word, protocol supplementary concept word, rare disease supplementary concept word, unique identifier] |  |
| 16. | 12 or 13 or 14 or 15 |  |
| 17. | exp Diet/ |  |
| 18. | exp Obesity/ or exp Obesity, Morbid/ or exp Obesity, Abdominal/ |  |
| 19. | (diet* or obes* or eating*).mp. [mp=title, abstract, original title, name of substance word, subject heading word, keyword heading word, protocol supplementary concept word, rare disease supplementary concept word, unique identifier] |  |
| 20. | 17 or 18 or 19 |  |
| 21. | exp Life Style/ |  |
| 22. | exp Health Behavior/ |  |
| 23. | (lifestyl* or life-styl* or life styl* or (health adj2 behav*)).mp. [mp=title, abstract, original title, name of substance word, subject heading word, keyword heading word, protocol supplementary concept word, rare disease supplementary concept word, unique identifier] |  |
| 24. | 21 or 22 or 23 |  |
| 25. | 7 or 11 or 16 or 20 or 24 |  |
| 26. | exp Loneliness/ |  |
| 27. | exp Social Isolation/ |  |
| 28. | (lonel* or (social* adj1 isolat*) or (social* adj1 alienat*) or (liv* adj1 alone) or solitar* or aloneness or solitud*).mp. [mp=title, abstract, original title, name of substance word, subject heading word, keyword heading word, protocol supplementary concept word, rare disease supplementary concept word, unique identifier] |  |
| 29. | 26 or 27 or 28 |  |
| 30. | (old* people or old* adult* or old* pop* or old age* or elderly or "pension* age").mp. [mp=title, abstract, original title, name of substance word, subject heading word, keyword heading word, protocol supplementary concept word, rare disease supplementary concept word, unique identifier] |  |
| 31. | exp "Aged, 80 and over"/ or exp Aged/ |  |
| 32. | 30 or 31 |  |
| 33. | exp epidemiologic studies/ |  |
| 34. | exp Statistics as Topic/mt [Methods] |  |
| 35. | exp Data Interpretation, Statistical/mt [Methods] |  |
| 36. | (cohort* or case* or longitud* or cross-section* or observational* or epidemiological or mixed method* or follow up* or multi-variat* or correlation*).mp. [mp=title, abstract, original title, name of substance word, subject heading word, keyword heading word, protocol supplementary concept word, rare disease supplementary concept word, unique identifier] |  |
| 37. | 33 or 34 or 35 or 36 |  |
| 38. | 25 and 29 and 32 and 37 |  |
| 39. | limit 38 to english language |  |
