# Supplementary material for: Loneliness and social isolation causal association with health-related lifestyle risk in older adults: a systematic review and meta-analysis protocol
Source: Syst Rev. 2019 Feb 7;8:48. doi: 10.1186/s13643-019-0968-x (PMC6366024; doi:10.1186/s13643-019-0968-x)
Supplement: Supplementary file 2 — Date Extraction Form Variables. File contains the details on the variables and their groupings to be extracted and recorded on the study data extraction form for the systematic review (DOCX 13 kb) [file 13643_2019_968_MOESM2_ESM.docx]

**Additional File 2: Data Extraction Form Variables**

| **General Study information** | Study ID/Surname/Year  Date data extracted  Name Reviewer extracting data  Reference citation (inc. authors, year, country, journal)  Notes |
| --- | --- |
|  | Type of publication |
|  |  |
| **Study eligibility** | Study type (observational, review, other)  Participants age |
|  | Exposure (loneliness/Social Isolation/both) |
|  | Measurement scale |
|  | Comparison type |
|  | Outcome (alcohol/smoking/drugs/obesity/physical activity/combination/other) |
|  | Outcome measure |
|  | Results reported |
|  | Status (include/exclude) |
|  | Exclusion reason (full text screened studies) |
|  |  |
| **Included Studies - Methods** | Study aim |
|  | Study design (i.e. cohort/cross-section/longitudinal) |
|  | Unit of observation |
|  | Start Date |
|  | End Date |
|  | Duration of participation |
|  |  |
| **Participants, outcomes & results** | Population description |
|  | Setting |
|  | Inclusion criteria |
|  | Exclusion criteria |
|  | Recruitment method |
|  | No. Of participants |
|  | No. Lost to follow-up |
|  | Non-response rate |
|  | Independent exposure variable |
|  | Subject characteristics reported in results 1-6 |
|  | Outcome 1 |
|  | Outcome 2 |
|  | Outcome 3 |
|  | Outcome 4 |
|  | Results 1 (primary outcome e.g. Odds ratio, risks ratio, hazards ratio, prevalence ratio) inc. Confidence intervals |
|  | Results 2-5 (secondary outcomes) |
|  | Results 2-5 (adjusted analyses) |
|  | Reported study limitations |
|  |  |
